# Role of Iron Indices in Anemia in Patients With Pulmonary Tuberculosis

**DOI:** 10.1155/ipid/2583917

**Published:** 2025-09-08

**Authors:** Bashir Abdrhman Bashir, Hagar M. Mohamed, Mohamed M. Hassan, Walaa Yasier Ali, Ehssan Moglad, Mohamed A. Hussain, Wadah Osman, Duaa Fahad Alsiyud, Gamal A. Mohamed, Sabrin R. M. Ibrahim

**Affiliations:** ^1^Department of Hematology, Faculty of Medical Laboratory Sciences, Port Sudan Ahlia University, Port Sudan 33312, Sudan; ^2^Department of Medical Laboratory Analysis, College of Medical & Health Sciences, Liwa University, Abu Dhabi 41009, UAE; ^3^Department of Applied Medical Chemistry, Medical Research Institute, Alexandria University, Alexandria, Egypt; ^4^Department of Hematology, Faculty of Medical Laboratory Science, National University-Sudan, Khartoum 11111, Sudan; ^5^Department of Applied Biology, Faculty of Sciences, University of Sharjah, Sharjah 211, UAE; ^6^Department of Pharmaceutics, College of Pharmacy, Prince Sattam bin Abdulaziz University, Al-Kharj 11942, Saudi Arabia; ^7^Department of Pharmaceutical Microbiology, Faculty of Pharmacy, International University of Africa, Khartoum 11111, Sudan; ^8^Department of Pharmacognosy, Faculty of Pharmacy, Prince Sattam bin Abdulaziz University, Al-Kharj 11942, Saudi Arabia; ^9^Department of Medical Laboratories-Hematology, King Fahd Armed Forces Hospital, Corniche Road, Andalus, Jeddah 23311, Saudi Arabia; ^10^Department of Natural Products and Alternative Medicine, Faculty of Pharmacy, King Abdulaziz University, Jeddah 21589, Saudi Arabia; ^11^Department of Chemistry, Preparatory Year Program, Batterjee Medical College, Jeddah 21442, Saudi Arabia

**Keywords:** anemia, health and wellbeing, iron deficiency, iron indices, Port Sudan, PTB, pulmonary tuberculosis

## Abstract

Iron indices are pivotal in tuberculosis (TB) owing to their influence on pathogens and immune reactions. Iron indices substantially affect TB progression, resulting in inflammation and anemia. Tuberculosis can induce iron deficiency or excess that may result in compromised immunological function. This study examined the iron index hemoglobin (Hb), serum iron, ferritin, total iron binding capacity (TIBC), unsaturated iron binding capacity (UIBC), and transferrin saturation (TSAT) in PTB patients. Between January 2016 and December 2018, the Port Sudan Tuberculosis Diagnostic Center studied a cohort of 100 adult patients definitively diagnosed with PTB. Additionally, 100 healthy individuals of similar age and sex were chosen as controls for comparative analysis. Among the 100 PTB patients studied, 90% (90/100) had anemia, with an odds ratio of 0.923 (95% CI 0.82–1.04). Anemia of chronic disease (ACD) was the most prevalent type (37%, 31/90). The patients showed diminished levels of HB, serum iron, TIBC, and TSAT compared to the controls, except for ferritin levels. UIBC was higher in patients than in controls, but this difference was not statistically significant. The research concludes that iron metabolism is modified during tuberculosis infection. Consequently, anemia in PTB patients is primarily attributed to ACD rather than iron shortage. The indices of serum iron, TIBC, and UIBC were ineffective in distinguishing between the forms of anemia in PTB patients, as their levels fluctuated in response to the infection. Ferritin served as superior metric for distinguishing between anemia of chronic disease and iron deficiency anemia.

## 1. Introduction

Tuberculosis (TB) is a prominent cause of morbidity and death globally, with an increasing number of cases being resistant to pharmacological treatment. According to a survey conducted by the World Health Organization (WHO) in 24 countries from 2007 to 2016, the percentage of confirmed cases of pulmonary tuberculosis (PTB) caused by bacteria ranged from 24% to 62% in African countries and from 33% to 68% in Asian countries [1–3]. The Sudan Health Observatory, associated with the Federal Ministry of Health, identifies malaria, TB, schistosomiasis, pneumonia, and diarrhea as the primary causes of morbidity among infectious diseases [4, 5]. Tuberculosis is estimated to account for 1% of all fatalities among hospitalized patients in Sudan in 2017 [6].

PTB prevalence in Sudan is a major public health issue. Thorough research has revealed that the aggregated PTB prevalence among several Sudan populations is approximately 30.72%. Their study included individuals from many states: Khartoum, Gezira, Kassala, Blue Nile, River Nile, White Nile, Gadarif, Red Sea, North Kordofan, Northern State, Sennar, and West Darfur [7]. Numerous factors, such as socioeconomic conditions, population density, and access to healthcare, affect TB prevalence of TB in eastern Sudan. Research in eastern Sudan, encompassing Port Sudan, revealed a tuberculosis notification rate of 275 per 100,000 individuals in 2012 [8]. Malnutrition and illness generate an adverse cycle that worsens each other. Malnutrition diminishes immune system performance, resulting in infection. Conversely, infection contributes to nutritional deficits. Iron is a crucial micronutrient for humans and pathogenic microorganisms. It functions as an enzyme cofactor and is indispensable for numerous cellular processes, such as respiration, DNA replication, oxygen transport, energy metabolism, and immunological excellence [9]. Iron deficiency results from insufficient iron in the body and often leads to anemia, which causes weakness and increases the risk of infection. Studies have shown that anemia is prevalent in 30%–94% of patients with tuberculosis [10]. Furthermore, compelling data indicate that anemia in patients with tuberculosis increases the likelihood of mortality [11]. According to a Port Sudan study, 15.3% of patients, especially those aged between 19 and 85 years, had iron deficiency anemia. In Port Sudan, 16% of PTB patients exhibit iron-deficiency anemia [12].

During TB infection, the body often exhibits hypoferremia (low serum iron) due to iron sequestration and decreased dietary absorption. This is an innate immune response to withholding iron from pathogens such as *Mycobacterium tuberculosis* [13]. Excessive iron in the host causes cellular toxicity through the iron-catalyzed production of reactive oxygen intermediates and hydroxyl radicals, damaging lipids, DNA, and proteins. The accumulation of iron in tissues and organs increases the likelihood of developing arthritis, cancer, liver disorders, diabetes, and heart failure. Elevated iron levels are associated with infectious diseases and inflammatory reactions such as malaria, viral infections, and neurodegenerative disorders [13]. TB is caused by *Mycobacterium tuberculosis*, which strictly depends on iron for its growth and reproduction. Within the framework of *Mycobacterium tuberculosis*, iron is taken up by siderophore-mediated uptake. Iron is vital for the development and multiplication of bacteria. Excess iron in *Mycobacterium tuberculosis* is risky because it catalyzes the formation of free radicals [14]. Iron biomarkers such as serum ferritin, total iron-binding capacity (TIBC), and transferrin saturation (TSAT) are correlated with the severity of PTB. Elevated serum ferritin coupled with diminished TSAT correlates with more severe manifestations of PTB, as evidenced by clinical evaluation [15]. This hypothesis posits that particular iron indices may function as biomarkers for evaluating PTB severity, thereby facilitating improved disease management and therapeutic approaches.

## 2. Materials and Methods

This case-control study was conducted at the Port Sudan Tuberculosis Diagnostic Center in Sudan, between January 2016 and December 2018. One hundred adult patients, comprising both males and females, who were confirmed to have PTB, were enrolled in the study based on statistical power. A group of 100 healthy volunteers matched for age, socioeconomic status, and lifestyle variables were designated as controls. These individuals were tested to confirm the absence of *Mycobacterium tuberculosis*, symptoms of TB or other lung diseases, and any contact with patients diagnosed with TB. Written informed consent was obtained from patients for their anonymized information to be published in this article.

Patients were excluded if they had multidrug-resistant (MDR) or extrapulmonary tuberculosis, a prior history of pulmonary TB, comorbidities that could alter iron metabolism or hematologic indices (e.g., significant renal, cardiac, or respiratory disease—including lung cancer—neoplasms, diabetes, and endocrine or genetic disorders), were currently receiving anti-tuberculous therapy or other drugs affecting bone marrow or peripheral blood, or had any chronic illness known to adversely affect bone marrow or peripheral blood function. Individuals who tested positive for HIV, pregnant or breastfeeding women, and those using oral nutritional supplements were also excluded from the study. These exclusion criteria were carefully crafted to guarantee the validity of the study. Individuals diagnosed with PTB for the first time and with no other chronic illnesses were included.

### 2.1. Study Method and Characteristics

Approximately 3 mL of blood was placed in a vacutainer containing tri-potassium ethylene diamine tetra acetic acid (K_3_EDTA), and another 3 mL was poured into a plain vacutainer. The samples were processed at typical laboratory temperature to extract serum via centrifugation. Expert technicians performed the study at the Port Sudan Tuberculosis Diagnostic Center.

The patients' features of interest were as follows: (1) demographic information, including the individual's sex, age, place of residence, tribe, and occupation. (2) Hematological measurements were performed using the cyanmethemoglobin method with a hematology analyzer (Mindray BC30s, China) to determine the hemoglobin concentration (Hb). (3) Chemical tests, including serum iron (lot; 145323008, linearity up to 1117 µg/dL, China), TIBC (Spinreact, lot; B225 linearity up to 1000 µg/dL, Spine), ferritin (lot; 2024070141, linearity up to 1500 μg/dL, China), unsaturated iron-binding capacity (UIBC) (lot; 146724003, linearity up to 558 μg/dL, China), and C-reactive protein (CRP) (lot; 146924021, linearity up to 250 mg/L, China) were analyzed within 2 h of collection using a fully automated biochemistry analyzer (Mindray BS200, China). TSAT was calculated. Chemical tests were conducted using Mindray reagents from China.

### 2.2. Determination of Sample Size

The sample size of 100 PTB patients and 100 matched controls were determined using standard epidemiological metrics, with a strong foundation in previous regional research. Given an anticipated anemia prevalence of 75% among PTB patients, a 95% confidence level, and an 80% power, the minimum necessary sample size was calculated using the formula for comparing proportions in case-control studies. An effect size of 0.3 and a two-tailed significance level (α) of 0.05 were used [16]. The sample size was adjusted to 100 per group to account for potential data loss and subgroup analysis.

### 2.3. Definitions for Iron Classifications

Absolute iron shortage is characterized by a significant decrease or absence of iron reserves in reticuloendothelial organs (ferritin < 20 μg/L).

Functional iron deficiencies are marked by an inability to effectively transport iron from the liver to other storage locations despite sufficient iron reserves (TSAT < 20; ferritin > 300 μg/L).

Iron blockade is an event in which iron is sequestered within macrophages during inflammatory responses.

Iron overload occurs when the body consistently absorbs excessive iron above its requirements (TSAT > 50; ferritin > 300 μg/L) [15].

### 2.4. Anemia Assessment

Anemia constitutes a worldwide public health concern impacting individuals of all ages in developing and underprivileged countries. Anemia is characterized by hemoglobin levels of 12.0 g/dL in females and 13.0 g/dL in males, as defined by the WHO. Anemia was categorized as follows: mild (11–11.9 g/dL), moderate (8–10.9 g/dL), severe (8 g/dL), and extreme (< 7 g/dL) for both males and females [17].

### 2.5. Statistical Analysis

Data entry was executed in SPSS, and analysis was completed utilizing Version 25 (Chicago, IBN, IL, USA). Categorical variables have been outlined by frequency (*n*) and percentage (%). Continuous variables were summarized using the mean and standard deviation. The Spearman rank correlation test was applied to analyze the interaction between continuous variables. The Kolmogorov–Smirnov test results indicated that the variables in each group did not conform to a normal distribution. The nonparametric Mann–Whitney and Wilcoxon signed-rank tests were conducted to compare the two groups. The disparities in hematological status and biochemical iron markers were examined via analysis of variance (ANOVA) with Tukey's multiple comparison test and the Kruskal–Wallis test. The prevalence and intensity of anemia were represented as a proportion with 95% confidence intervals. Variables identified through bivariate analysis were incorporated into the multivariable log-binomial regression model to ascertain the significance associated with hematological issues. All analyses assigned significance to *p* < 0.05.

## 3. Results

The baseline characteristics of the study population are presented in Table 1. Of the 100 PTB patients, 77% were male and 23% were female. The average age of the PTB patients was 32.9 ± 12.5 years. For comparison, the 100 healthy subjects included 76% were males and 24% were females, with an average age of 27.1 ± 9.4 years. Age and iron indices were significantly different between the PTB patients and controls (Table 1).

Table 1 displays contrasting features of the patients and controls. The data indicated that the southern section of the study area, Darussalam, had the highest prevalence of tuberculosis infection, accounting for 52% of the cases. Workers were the most commonly affected occupational group, accounting for 42% of the cases. The Western tribe had the highest percentage of pulmonary TB cases (35%), followed by the Bani Amer tribe (33%), Hadandwa tribe (24%), and northern tribe (8%).

Significant differences in HB, iron levels, TIBC, ferritin levels, and TSAT levels were detected between the patient and control groups. The patient group exhibited reduced levels of hemoglobin, serum iron, TIBC, and TSAT compared to the control group, except for ferritin levels (*p* ≤ 0.001) (Table 1). The UIBC was also elevated in the test group compared to the control group, although this difference was not statistically significant (0.887).

### 3.1. Epidemiology of Anemia

Anemia was detected in 90.0% of patients with PTB (95% CI, 75%–107%), with an odds ratio of 0.923 (95% CI 0.82–1.04). The intensity of anemia was categorized as follows: mild anemia, 34.4% (95% CI 19.8%–47.3%); moderate anemia, 17.8% (95% CI 10.3%–24.5%); severe anemia, 31.1% (95% CI 17.9%–42.8%); and extreme anemia, 16.7% (95% CI 9.6%–23.0%). The overwhelming majority of patients experienced a mild 34.4% and severe anemia (31.1%).

**Figure 1:**
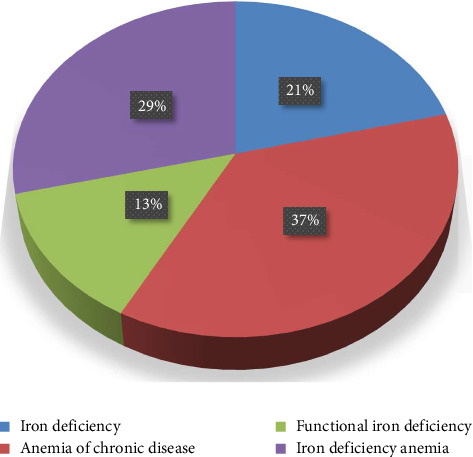
This chart illustrates the categories of iron states within the study.

Approximately 68% of patients diagnosed with anemia were males. Among the people who tested, the most common type of anemia was ACD, accounting for 37% (33 out of 90 cases). Subsequently, IDA occurred in 26/90 patients (29%). Iron deficiency was ranked as the third most prevalent, accounting for 21.1% of the 19/90 cases. Functional iron deficiency anemia accounted for 12/90 cases (13.3% of the patients with anemia) and was the least prevalent form (Figure 1).

**Figure 2:**
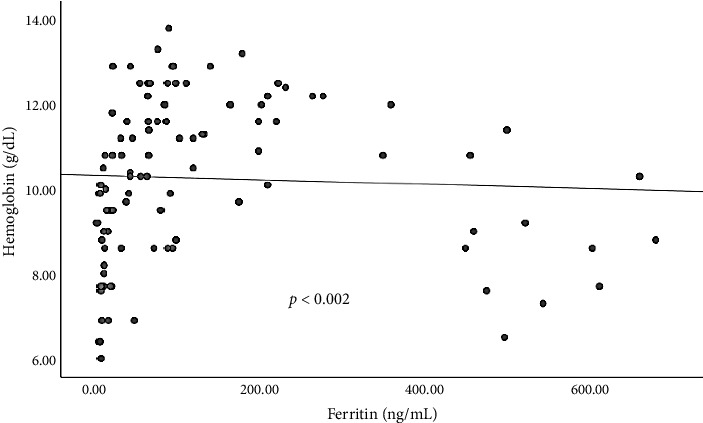
This curve demonstrates the substantial correlation between ferritin and hemoglobin in patients with PTB.

Sex was significantly associated with TIBC and UIBC (*p* < 0.005 and 0.027, respectively) but was not significantly associated with HB, iron, ferritin, and TSAT (*p* < 0.514, 0.104, 0.384, and 0.232, respectively). No significant association was observed between sex and age in this study (*p* < 0.915). This study showed a substantial association between ferritin and hemoglobin levels in patients with PTB (*p* < 0.002) (Figure 2).

### 3.2. Outcomes of the Study

**Figure 3:**
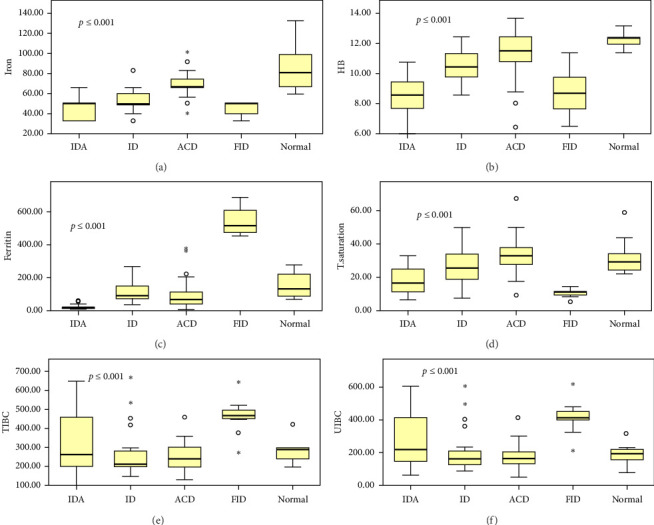
Box plot illustrates the pattern of distribution of biochemical parameters among the PTB patients. A *p* value < 0.05 is deemed statistically significant, (a) iron, *p* ≤ 0.001; (b) HB; (c) ferritin; (d) transferrin saturation; (e) total iron-binding capacity; (f) unsaturated iron-binding capacity; *p* ≤ 0.001.

In this study, iron concentrations were strongly associated with ACD (*p* ≤ 0.001, 95% CI 15.0–35.3). TSAT values demonstrated statistical significance with ACD (*p* ≤ 0.001, 95% CI 7.1–23.3). , TSAT values were significant with FID (*p* ≤ 0.001, 95% CI 12.5–23.2). As a result, ferritin levels were statistically significant in ACD (*p* ≤ 0.001, 95% CI 23.4–128.2). Ferritin levels also proved significant with FID (*p* ≤ 0.001, 95% CI 376.3–511.1). The present study revealed that iron parameters were statistically significant across various types of anemia, namely, ACD, IDA, ID, and FID (*p* ≤ 0.001) (Figure 3). Interestingly, the tribe was positively correlated with HB, ferritin, and TSAT levels (*r* = 0.443; *p* ≤ 0.001, *r* = 0.154; *p* < 0.030, *r* = 0.146; *p* < 0.039, respectively).

In PTB patients, HB was positively correlated with iron, ferritin, and TSAT levels (*r* = 0.663; *p* ≤ 0.001, *r* = 0.507; *p* ≤ 0.001, *r* = 0.351; *p* ≤ 0.001, respectively) and negatively correlated with sex, age, TIBC, and UIBC (*r* = −0.162, *p* < 0.022, *r* = −0.236; *p* ≤ 0.001, *r* = 0.030; *p* < 0.670 and *r* = −0.096; *p* < 0.177, respectively). Iron levels were significantly correlated with age, HB, ferritin, and UIBC (*r* = 0.177; *p* < 0.012, *r* = 0.663; *p* ≤ 0.001, *r* = 0.269; *p* ≤ 0.001, *r* = 0.240; *p* < 0.031, respectively), and showed a low correlation with TIBC and TSAT (*r* = 0.025; *p* < 0.725, *r* = −0.629; *p* ≤ 0.001, respectively). In addition, TIBC was negatively correlated with TSAT and weakly positively correlated with ferritin (*r* = −0.700; *p* ≤ 0.001, *r* = 0.151; *p* < 0.033).

## 4. Discussion

Both infections and the host require iron for their survival [18]. Pathogens utilize iron to thrive within their hosts by competing for intracellular iron or synthesizing compounds with a high affinity for iron [19]. Investigating the correlation between anemia and iron indices is a challenging task. This study assessed the prevalence and iron indices related to anemia among PTB patients in Eastern Sudan. Nearly 90% of those with PTB had anemia, with 66% experiencing mild or severe forms of the condition. According to the WHO classification, 90.0% of the patients with PTB in our study presented with anemia at the time of diagnosis. The prevalence reported in studies from India by Mukerjee et al. (71.8%) and Dileepan et al. (75.5%), as well as from Indonesia by Sahiratmadja et al. (63%), is consistent with our findings [8–21].

The significant prevalence of anemia among patients with TB warrants public attention. Anemia negatively affects tuberculosis in multiple ways. Data from the general population indicate that anemia correlates with all-cause mortality irrespective of age, sex, and cardiovascular disease [22]. Anemia, with or without iron deficiency, is consequently linked to a three-fold increase in mortality risk among individuals with tuberculosis [11]. Anemia in tuberculosis and the accompanying systemic inflammation do not necessarily resolve with tuberculosis treatment and may present risks for further problems, even postcure. Consequently, there is a need to implement population-wide interventions to reduce the impact of anemia on PTB as part of efforts to decrease the incidence of PTB. Furthermore, protocols for managing anemia in PTB are essential to enhance the treatment outcomes in patients with anemia. Moreover, longitudinal studies are imperative to further delineate the progression of anemia and its potential consequences following PTB resolution [23–25].

Hb levels decreased among patients with anemic PTB. The average HB readings were 10.3 g/dL in anemic participants and 13.4 g/dL in nonanemic subjects, consistent with findings from other Indian investigations [20]. Hematological alterations that develop are frequently linked to the immunological response of the body to tuberculosis infection. A drop in hemoglobin levels in anemic tuberculosis patients may be associated with the intensity of tuberculosis infection and inflammation, potentially affecting erythropoiesis and resulting from iron scarcity [26].

Despite the anemia, serum iron and TSAT indices were diminished in all PTB patients, whereas ferritin and UIBC levels were elevated. A similar observation was reported by Bashir et al. [27]. Our investigation revealed significant positive relationships between ferritin, serum iron, TSAT, and Hb, indicating a marginal increase in Hb as the iron indices increased. The restricted correlation may arise from the inflammation associated with PTB. Increased ferritin and TIBC levels may inhibit the proliferation of *Mycobacterium tuberculosis* by restricting iron availability in response to infection [28]. Ferritin levels are diminished in IDA. At the same time, inflammation can exacerbate these conditions, similar to serum iron and TIBC, complicating the interpretation in iron-deficient patients with concurrent infections or inflammation. Patients with PTB commonly demonstrate higher plasma ferritin levels, which may signify oxidative stress [28]. This unequivocally confirmed functional iron insufficiency was observed in our patients (13%).

In the current study, ACD was the most prevalent type of anemia, in contrast to IDA (33 [37.0%] vs. 26 [29.0%]). Other researchers, including Mario Oliveria (75.9%), Minchella et al. (36%), Bashir A (34%), and Hella et al. (59.8%), identified ACD as the predominant type of anemia among PTB patients globally. The reported proportions exhibit significant variability, likely due to differing definitions that distinguish ACD from IDA [12, 23, 28–30].

ACD results from chronic diseases marked by a continual inflammatory state, which causes inflammation-related complications such as diminished erythrocyte lifespan, poor iron absorption in erythrocytes, and reduced response to or possession of erythropoietin [30]. IDA arises solely from insufficient nutritional intake and malabsorption, resulting in food instability and diminished appetite in PTB patients. IDA and ACD-related IDA arise from disturbances in iron equilibrium, with the latter frequently associated with inflammation [31, 32]. Our analysis also identified a limited number of cases of iron deficiency. Chronic diseases complicate the interpretation of diagnostic iron measurements, including ferritin, serum iron, TIBC, and TSAT [32].

The correlation analysis identified multiple significant correlations among the study variables. Tribes were positively correlated with hemoglobin, iron, and ferritin levels, suggesting that genetic or dietary influences may influence these hematological parameters [33]. Age showed substantial negative correlations with hemoglobin, iron, and tribe levels, indicating that hemoglobin and iron levels diminish with age. This aligns with the literature finding that aging is frequently linked to declines in hematological health [34]. Sex had a strong negative association with age, hemoglobin, and ferritin. Females may have lower hemoglobin and ferritin levels than males, which is consistent with research emphasizing sex disparities in hematological parameters [35].

Hemoglobin (Hb) exhibited a robust positive correlation with blood iron (*r* = 0.663, *p* < 0.001), indicating that elevated iron levels correlate with enhanced hemoglobin synthesis. Furthermore, hemoglobin (Hb) was positively correlated with TSAT (*r* = 0.351, *p* < 0.001) and ferritin (*r* = 0.507, *p* < 0.001), indicating that elevated iron reserves and iron transport ability are associated with increased hemoglobin concentrations [36–38]. TIBC was significantly negatively correlated with TSAT (*r* = −0.700, *p* < 0.001). This signifies that an increase in the total iron-binding capacity corresponds to a decrease in iron-saturated transferrin. This inverse association underscores the dynamic interaction between iron availability and binding capacity [39]. TIBC showed a weak positive correlation with ferritin (*r* = 0.151, *p* < 0.001), indicating a possible association between iron-binding capacity and iron storage. These findings offer significant insights into the intricate link between iron metabolism and hemoglobin synthesis in the context of PTB, a discovery that carries significant weight in the fields of healthcare and research.

Hepcidin and erythroferrone are crucial regulators of iron metabolism, particularly during inflammatory conditions such as tuberculosis. Hepcidin, made up by the liver, impedes iron absorption and release by destroying ferroportin. In persistent infections, increased hepcidin exacerbates anemia of inflammation by sequestering iron within macrophages and diminishing serum iron availability [40]. Erythroferrone, synthesized by erythroblasts in response to erythropoietin, inhibits hepcidin to facilitate iron mobilization during heightened erythropoietic demand. In tuberculosis, the equilibrium between these hormones is disrupted, resulting in a functional iron deficiency despite sufficient iron reserves [41].

Due to the inflammatory characteristics of TB-associated anemia, anti-inflammatory treatments may enhance conventional TB treatment. Agents that target IL-6 or hepcidin pathways, such as hepcidin antagonists or erythroferrone mimetics, may enhance iron availability and promote erythropoiesis. Nutritional therapies, such as iron supplementation, must be judiciously evaluated based on ferritin and TSAT levels to prevent the aggravation of infection or oxidative stress [42].

The strength of this study lies in the integration of iron clues to characterize various types of anemia, distinctly differentiating ACD from iron deficit and functional iron deficiency. This study had some limitations. Patients originate from urban locations, and the levels of inflammation may differ from one area to another. The patients' potential sources of inflammation, apart from tuberculosis, have not been explored. The levels of soluble transferrin receptors are recommended for further research.

## 5. Conclusion

Anemia was highly prevalent among patients with proven PTB at the Port Sudan Tuberculosis Diagnostic Center in Sudan. Most patients exhibit mild-to-severe anemia. Anemia was correlated with characteristics indicative of severe tuberculosis. The iron, TIBC, and TSAT levels were lower in the patient group than in the control group. Conversely, UIBC and ferritin levels were elevated in PTB patients. Elevated ferritin levels do not indicate increased iron reserves. Ferritin correlates closely with clinical severity and may be a valuable marker of disease activity and mortality risk. Strategies for managing anemia in tuberculosis are essential in low-income settings, where resources for determining the precise etiology of anemia are limited.

## Figures and Tables

**Table 1:** Overview and biochemical analysis of the study's population.

Characteristics	PTB patients (*n* = 100)	Controls (*n* = 100)	*p* value
Age (mean ± SD)	32.9 ± 12.5	27.1 ± 9.4	≤ 0.001
(Range)	14–70	19–63	
Sex			0.897
Male	77 (77%)	76 (76%)	
Female	23 (23%)	24 (24%)	
Demographic data			
Residence			0.073
Eastern area	32 (32%)	48 (48%)	
Southern area	52 (52%)	36 (36%)	
Downtown	16 (16%)	16 (16%)	
Tribes			≤ 0.001
Hadandwa	24 (24%)	12 (12%)	
Bani Amer	33 (33%)	14 (14%)	
Northern Sudan	8 (8%)	58 (58%)	
Western Sudan	35 (35%)	16 (16%)	
Occupation			≤ 0.001
Student	11 (11%)	50 (50%)	
Workers	42 (42%)	24 (24%)	
Housewife	13 (13%)	—	
Employees	14 (14%)	26 (26%)	
Other jobs	20 (20%)	—	
Biochemical parameters			
Hemoglobin (g/dL)	10.3 ± 1.9	13.4 ± 1.5	≤ 0.001
Serum iron (μg/dL)	60.0 ± 19.2	90.5 ± 21.8	≤ 0.001
Serum TIBC (μg/dL)	301.0 ± 137	312.7 ± 83.2	≤ 0.001
Serum UIBC (μg/dL)	241.0 ± 143	220.0 ± 85.5	0.887
Serum ferritin (ng/mL)	136.9 ± 169	120.8 ± 13.3	≤ 0.001
TSAT (%)	24.9 ± 13.3	31.9 ± 11.9	≤ 0.001

## Data Availability

The data will be made available at reasonable request to corresponding authors.
